# American Sign Language Recognition and Translation Using Perception Neuron Wearable Inertial Motion Capture System

**DOI:** 10.3390/s24020453

**Published:** 2024-01-11

**Authors:** Yutong Gu, Hiromasa Oku, Masahiro Todoh

**Affiliations:** 1 Faculty of Informatics, Gunma University, Kiryu 3768515, Japan; h.oku@gunma-u.ac.jp; 2 Graduate School of Engineering, Hokkaido University, Sapporo 0608628, Japan; 3 Faculty of Engineering, Hokkaido University, Sapporo 0608628, Japan; todoh@eng.hokudai.ac.jp

**Keywords:** American sign language, wearable inertial sensors, deep learning models

## Abstract

Sign language is designed as a natural communication method to convey messages among the deaf community. In the study of sign language recognition through wearable sensors, the data sources are limited, and the data acquisition process is complex. This research aims to collect an American sign language dataset with a wearable inertial motion capture system and realize the recognition and end-to-end translation of sign language sentences with deep learning models. In this work, a dataset consisting of 300 commonly used sentences is gathered from 3 volunteers. In the design of the recognition network, the model mainly consists of three layers: convolutional neural network, bi-directional long short-term memory, and connectionist temporal classification. The model achieves accuracy rates of 99.07% in word-level evaluation and 97.34% in sentence-level evaluation. In the design of the translation network, the encoder-decoder structured model is mainly based on long short-term memory with global attention. The word error rate of end-to-end translation is 16.63%. The proposed method has the potential to recognize more sign language sentences with reliable inertial data from the device.

## 1. Introduction

Sign language is the primary communication method among deaf and hard-of-hearing people. According to the World Health Organization, nearly 2.5 billion people are projected to have some degree of hearing loss, and at least 700 million will require hearing rehabilitation by 2050 [1]. With the development of artificial intelligence technology, more and more deep learning models appear in natural language processing (NLP), bringing about significant changes in human society. Therefore, it is critical to apply deep learning models to promote the research on sign language translation.

Prior work of sign language recognition can be divided into the following two main mechanisms: vision-based and wearable sensors-based recognition. The vision-based approach utilizes RGB or RGB-D cameras to catch the dynamic movements of the hands [2,3]. Some of the commonly used corpora containing a large amount of data are RWTH-PHOENIX-Weather [4] and Chinese Sign Language (CSL) [5]. The RWTH-PHOENIX-Weather selects the sign language interpretation of daily news and weather forecasts over three years. The CSL dataset collected by Kinect contains 100 continuous Chinese sign language sentences.

Compared with vision-based methods, the data amount of wearable sensors-based corpora is generally small due to limited data sources. The MyoSign [6] covering 70 commonly used American sign language (ASL) words and 100 ASL sentences incorporated multimodal Convolutional Neural Network (CNN), bidirectional Long Short Term Memory (LSTM), and Connectionist Temporal Classification (CTC) to achieve continuous sign language recognition. The sentences were stitched together by the gestures of 70 words, but not coherent actions of sign language performance. The SignSpeaker [7] deployed on a smartwatch and a smartphone to realize real-time, robust, and user-friendly sign language recognition. One hundred and three common-used words covering the diversity of the ASL actions were selected to generate 73 sentences, following the grammar of ASL. WearSign [8] leveraged a smartwatch and an armband to capture sophisticated sign gestures. In addition, a multi-task encoder-decoder framework was introduced to realize the end-to-end translation of 250 ASL sentences.

Wearable devices commonly used for sign language recognition include independent Electromyography (EMG) & inertial measurement unit (IMU) sensors, armbands, and data gloves. The Myo armband is a lightweight off-the-shelf wearable device providing nine inertial and eight-channel EMG signals. Many studies on sign language word and gesture recognition have used this device considering the portability of the armband [9,10,11,12,13]. Savur & Sahin [14] classified the gestures of 26 English alphabet letters with Myo armband. Jane & Sasidhar [15] did the classification of 48 sign language words. Paudyal et al. [16] achieved an accuracy of 97.72% for 30 ASL gestures. In addition to the armband, independent EMG sensors have been used in various studies [17,18]. Yu et al. [19] applied four EMG sensors and one inertial sensor spreading around the forearm to recognize 150 CSL subwords by the deep belief net. With a fusion strategy for combining multi-sensor and multi-channel information, Li et al. [20] developed a 121 CSL subwords recognition system with two 3-dimensional accelerometers (ACC) and eight EMG sensors worn on the forearm. It is also a novel idea to customize data gloves [21,22]. Feng et al. [23] utilized novel triboelectric textile sensors attached to the glove to measure hand gestures. Then, the glove signals were used to recognized 50 words and 20 sentences with the deep learning model. Korzeniewska et al. [24] presented a data glove using textronic elements produced by a physical vacuum deposition process. The ASL alphabet recognition accuracy of this device was 86.5%. Other wearables which are also suitable for hand gesture recognition include smartwatches, smart rings, and acoustic devices. Liu et al. [25] combined a smartwatch worn on the wrist and a smart ring worn on the index finger to recognize the 100 most frequently used ASL finger gestures. The accuracy was 94.2%. In addition, SonicASL [26] leveraged dual speakers and microphones to capture the sonic feedback from hand gestures. Given 42 frequently used ASL words and 30 sentences, the system could achieve an accuracy of 93.8% at the word level, and 90.6% at the sentence level. The summary of previous studies is listed in Table 1.

In the above-mentioned studies on sign language recognition using wearable sensors, more studies focused on single gestures. Few datasets for sentence recognition were confined to a limited number of sentences and did not consider grammatical inconsistencies between sign language and spoken language. Datasets containing more sentences for the end-to-end translation had a limited number of words in the vocabulary. The selected words restricted more natural expressions. Based on the experience of previous research, this study introduces a larger dataset containing 300 commonly used ASL sentences. Without deliberate selection, these sentences are composed of 455 different hand gestures. Two kinds of labels under the grammar of both sign language and spoken language are added to each sentence. Thus, two NLP models for both sequence recognition and end-to-end translation are also introduced.

The rest of the paper is organized as follows: Section 2 introduces the dataset collection process and the structure of deep learning models. After training the models with the collected data, Section 3 presents the results and evaluation of the models’ outputs. Then, the user-independent validation and the rationality of the methodology are discussed in Section 4. Finally, the conclusion of the paper is drawn.

## 2. Materials and Methods

The grammar of sign language can be different from the spoken language. For example, the expression “What’s your name” in ASL is the signs’ sequence of “your”, “name”, and “what”. It is also critical to emphasize the subject in declarative or interrogative sentences. When expressing “I am happy”, people tend to sign “I”, “happy”, and “I” to emphasize the protagonist of the topic at the end of the sentence. In this research, we prepare two kinds of labels for one sign language expression. One is under the order of hand gestures, and another is under the rule of spoken language.

**Figure 1:**
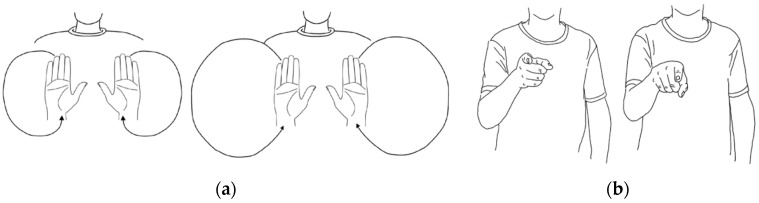
Different expressions for the same word: (**a**) “Sunday”; (**b**) “you”.

Except for grammar, the range of hand movements varies from person to person. In Figure 1a, the expressions of “Sunday” differ in the motion trajectories. Also, people can hardly keep the same movements for one specific word appearing in different sentences. Because the other words around that specific word are not the same, the gesture’s starting state (the ending state of the last word) can be different. One sign language word may also have similar but not identical expressions. As shown in Figure 1b, both gestures are for the word “you”, but they are slightly different in angle.

To solve the problem mentioned above, in this section, the motion capture system is utilized to collect the inertial data of arms and hands during sign language performance. Two deep learning models are introduced for sign language recognition and end-to-end sign language translation.

### 2.1. Dataset Collection

**Figure 2:**
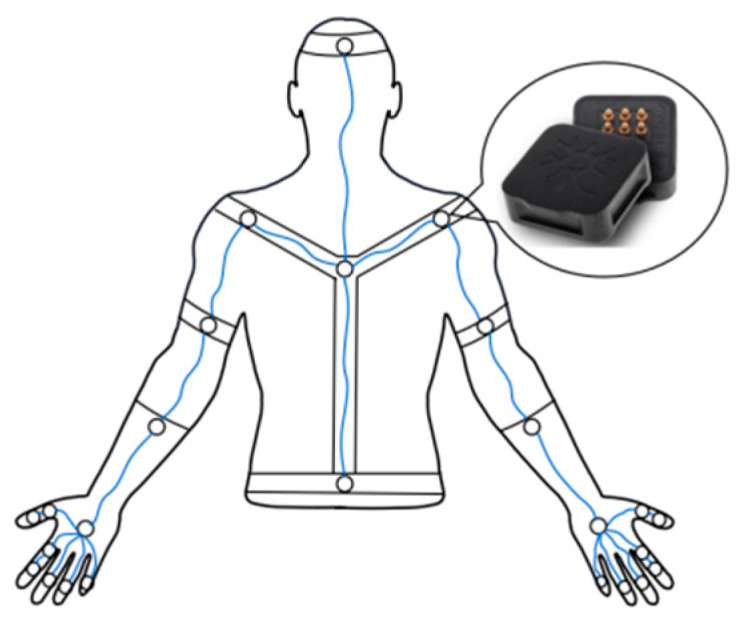
Sensor distribution under the human back view [27].

The upper-body movement is captured by the Perception Neuron (Noitom Ltd., Beijing, China) motion capture system. It includes 25 inertial measurement units (IMUs) fixed by fabric and straps spreading around the back, head, arms, and hands. The distribution of the IMU sensors is shown in Figure 2. Each sensor consists of an accelerometer, gyroscope, and magnetometer and has a small mass and size. Therefore, the device maintains a very light mass overall, even with the use of many sensors. Moreover, comprehensive human movement information is captured by the device and the data quality is high. Without the use of metal parts, the device doesn’t impede the body’s movement and is well-suited for wearing.

**Figure 3:**
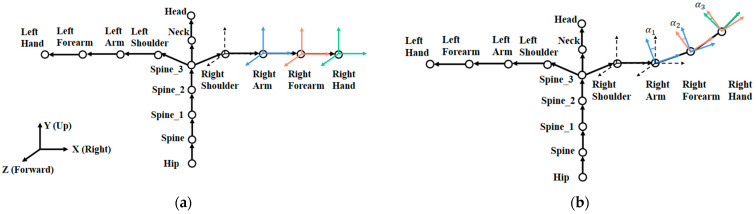
The relative relationship of upper-body joint coordinates: (**a**) initial sensor calibration posture; (**b**) right arm slightly raised.

**Figure 4:**
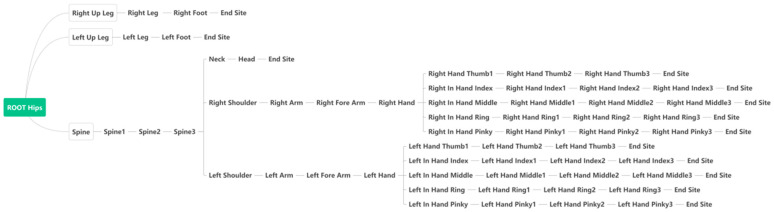
The hierarchy of all joint coordinate data returned by the device.

The device transfers data to the computer via USB or Wifi. Basic settings and sensor calibration are performed on the supporting software before use. The length of bones between the joints of the human body is considered to be fixed by selecting the user’s height at first. The sampling rate is 60 Hz. At each sampling moment, the data returned by the device is the calculated rotation data (Rotation_Y, Rotation_X, Rotation_Z) of each upper-body joint. The hip joint is a reference point recording the absolute coordinates under the earth coordinate system. The coordinates of other joints are relative coordinates based on their respective reference joints. Figure 3a shows the reference relationship between the coordinates (except for the hands) when performing the initial sensor calibration. The body is in one plane with both arms stretched horizontally. Taking the right arm as an example, the Right Arm takes the Right Shoulder as the reference, and the coordinate values indicate the difference in rotation angle around the three axes between the two. The same relationship exists between other joints. The coordinates of the Right Arm, Right Forearm, and Right Hand in this state are 0. In the next frame, the right arm is slightly raised leading to the positional change in the XOY plane, as shown in Figure 3b. The three mentioned joints produce a rotation around the Z-axis. The coordinate values of Rotation_Z become $\alpha_{1}$, $\alpha_{2}$, and $\alpha_{3}$ in the current frame. The relative information of the hand joints is added in Figure 4.

The device captures the rotation data of 59 upper-body joints, but only a part of them are helpful. Due to the limitation of the device, some finger joint movements only have data in the direction of extension/flexion. Moreover, some coordinates of joints (like hip, head, neck, and spine) keep the same value throughout the experiment because of the standing still status during the sign language performance. We manually remove these useless data, and the remaining coordinates are listed in Table 2. Finally, a 38-dimensional vector is selected to describe the state of arms and hands at each moment.

Through the survey of sign language online courses, 300 ASL sentences (some examples are listed in Table A1), including the basic topics of everyday life (weather, daily routine, age, etc.) are selected as the target sentences. Three ASL beginners (female, age: 23~29, height: 158~163 cm, weight: 43~50 kg) joined the experiment after taking the online courses. The data collection process lasted a month. Participant 1 contributed the data for all 300 sentences. Participants 2 and 3 contributed the data for 50 and 20 sentences, respectively. Each sentence was required to be repeated 20 times. Finally, the data amount of the collected ASL dataset was 7400.

Each sentence of sign language lasts for around 1~7 s. Under the sampling rate of 60 Hz, the data along the time direction contains 60~420 points. In the preprocessing of collected inertial data, a median filter is applied to make the data smooth. Then, the data is segmented into frames using the sliding window method. The window size is 36, and the sliding size is 18.

### 2.2. Model Structure

#### 2.2.1. Sign Language Recognition Model

**Figure 5:**
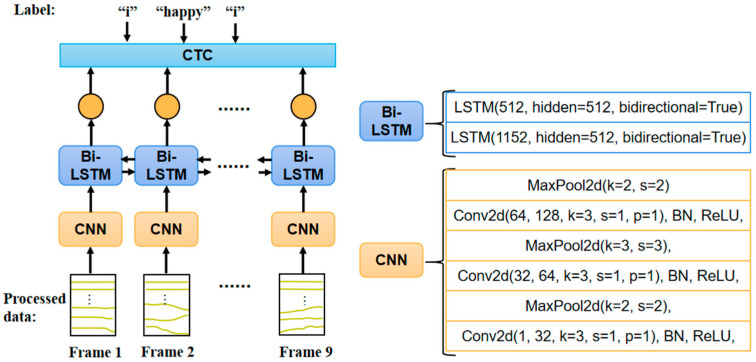
The sequence recognition model for the sign language recognition task.

A sign language sentence is made up of a series of hand gestures. In the sign language recognition task, the label is a series of words sharing the same order with hand gestures. So, hand gestures are required to be recognized correctly in order. In the sequence recognition model of Figure 5, the first layer is the CNN that extracts features from each input data frame. Based on shared weights architecture, CNN eliminates effects from motion differences in amplitude and trajectory [28]. The second layer is Bi-directional Long-Short Term Memory (Bi-LSTM). LSTM can preserve long-term dependencies by controlling the percentage of previous information dropping, current information inputting, and current information outputting [29]. In addition, Bi-LSTM utilizes both forward and backward information on each time step to better contextualize the output. By using the sliding window method in data preprocessing, the output sequence from the Bi-LSTM layer is much longer than the number of words in the label. Therefore, a CTC layer is added to realize the alignment between the output predictions and the label [30]. When using CTC as the loss to train the model, it calculates the sum of probabilities of all possible alignments.

(1) loss=−log∑p(alignment|input).

Although not only one alignment leads to the correct result, the output with the largest probability of each frame is chosen as the final result when doing the model validation.

In the preprocessing of text labels, a vocabulary with 455 words (listed in Table A2) for the 300 sentences is built. Thus, by looking up the vocabulary, a word can be represented by the index. Finally, a sentence is converted into a string of numbers in the range of 0~454.

#### 2.2.2. End-to-End Sign Language Translation Model

**Figure 6:**
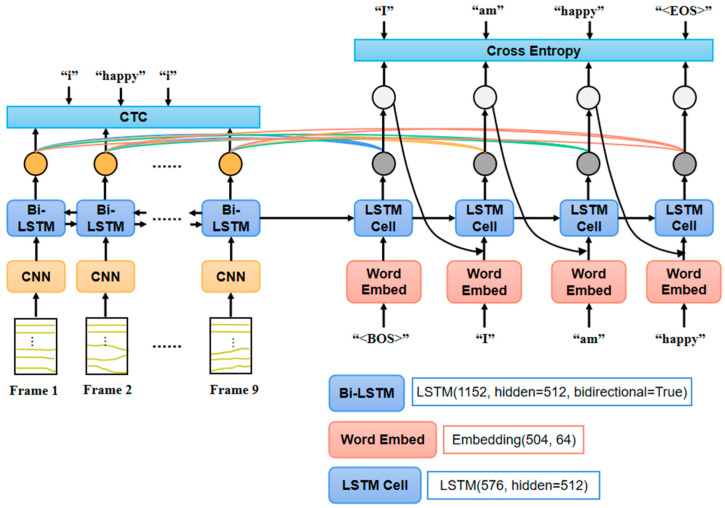
The encoder-decoder structured model for the sign language translation task.

In the end-to-end sign language translation task, the label sentence has the same grammar rule as the spoken language in daily life. However, some words like “am”, “is”, and “to” do not have corresponding expressions in sign language. Inspired by neural machine translation, an encoder-decoder structured model with global attention is applied to realize the end-to-end sign language translation. As shown in Figure 6, the encoder keeps nearly the same structure as the model in Section 2.2.1. The decoder consisting of the LSTM cell continues to output decoded words until the “end” marker is reached. Global attention between the encoder and decoder is added to learn the mutual mapping relationship between inertial data and the text sentence [31]. In the training step of the model, the input to the decoder in each time step is the combination of the real label word and the result of the last time step. The output of the current time step is determined by the output of the LSTM cell and the attention result from the encoder. The loss of the decoder is the cross entropy between the output distribution and the label word. The CTC loss from the encoder is also considered together to optimize the model.

The translation task’s vocabulary differs from the above-mentioned sequence recognition, so another vocabulary (listed in Table A3) is built for the label sentences under the spoken language grammar. In addition to the 502 ordinary words, two special tokens “<BOS>” and “<EOS>” representing “beginning of sentence” and “end of sentence” are also added to the vocabulary. All 300 sentences have these two special tokens added at the beginning and the end.

## 3. Results

### 3.1. Sign Language Recognition Results

The collected 7400 sentences are randomly divided into five equal parts. To do five-fold cross-validation, one part is taken as the validation set each time, and the other four parts are collectively used as the training set. The training set contains 5920 sentences and the validation set contains 1480 sentences. The deep learning model is built with Python 3.7 (Python Software Foundation, Beaverton, OR, USA) and PyTorch 1.13.1 (Meta AI, New York City, NY, USA). In the training step, the optimizer is Adam, with a learning rate of 0.0001. After training the model for 20 epochs, the CTC loss drops to a low level. The recognition results of the model on the validation set are listed in Table 3. The word error rate (*WER*) measures the least operations of substitution, deletion, and insertion to transform the predicted sequence into the label sequence.

(2) $$WER=\frac{N_{sub}+N_{del}+N_{ins}}{N_{all\;words}},$$

where *N_sub_*, *N_del_*, and *N_ins_* are the numbers of necessary substitutions, deletions, and insertions, respectively. The sentence error rate (*SER*) measures the percentage of not entirely correct sequences of the validation results.

(3) $$SER=\frac{N_{error\;sequences}}{N_{all\;sequences}}$$

When treating the sign language sentence as a sequence of hand gestures, the model can recognize each sequence with a high accuracy rate. In addition, the alignments between the model output and the label words are also well achieved. This shows that the data collected by the device can accurately represent different gestures.

### 3.2. End-to-End Sign Language Translation Results

The end-to-end translation model is trained with the same settings as the above-mentioned model. The error rates for both the word and sentence-level evaluation increase considerably in the end-to-end translation results, as illustrated in Table 4. Since the sentences outputted by the decoder tend to reach the “<EOS>” token earlier, the output sentences from the model are generally shorter than the label sentences, leading to a large number of insertion errors. Spoken language words that could not be expressed by hand gestures also show higher error rates in both substitution and insertion. Due to the small number of text sentences, the model could learn limited knowledge about English grammar, providing a limited enhancement effect on the translation results.

The end-to-end translation model utilizes both CTC loss and cross-entropy loss, and the labels for each type of loss come from different vocabularies. To verify the role of CTC loss, validation fold 4 which is closest to the average result is selected as the validation set. Then, the model is retrained after removing the CTC loss. From the results of the validation set, both WER and SER increase by 1.49% and 0.27%, respectively. This indicates that CTC loss promotes model optimization. 

**Figure 7:**
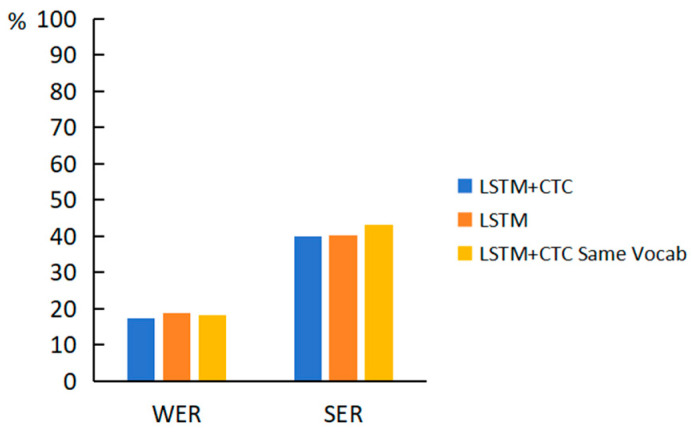
Comparative results of translation under different conditions.

The recognition and translation have two different vocabularies because the words that make up the two kinds of labels are not the same. When the two vocabularies are unified into one vocabulary, word indexes are also modified. When the model is trained with the modified labels, the resulting error rates are also increased by 0.97% and 3.18% compared to the original results, as shown in Figure 7. Using only one vocabulary causes the labels of CTC loss to be sparse, and many words in the vocabulary do not appear on CTC labels. This makes alignment more difficult, leading to an increase in error rates.

## 4. Discussion

### 4.1. User-Independent Validation

#### 4.1.1. Word-Level User-Independent Validation

Participants 2 and 3 only attended a part of the experiment (contributing 50 sentences and 20 sentences, respectively), and the sentences they contributed were quite different from each other. So, 20 words (listed in Table 5) for which all three participants have data recorded are selected for word-level user-independent validation. The dataset of these words is manually segmented from the sentences. The same word may come from different sentences, so the starting and ending states of the gestures for the same word could be different.

**Figure 8:**
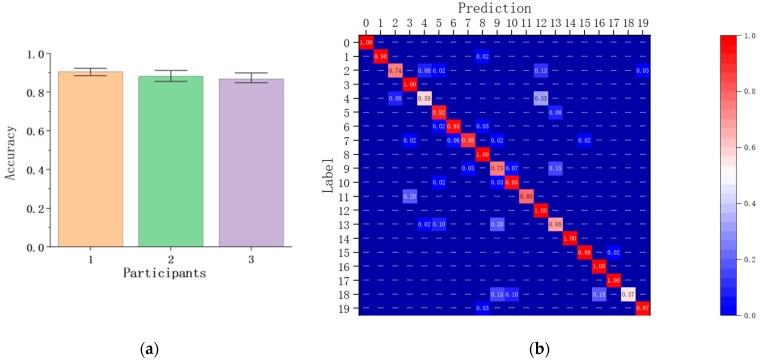
Word-level user-independent validation results: (**a**) Classification accuracy of three participants; (**b**) Confusion Matrix of all 20 words.

The classifier consists of the CNN feature extractor and a fully connected classification layer. The word data from one participant is used as the validation set, and the remaining data from the other two participants is used to train the classifier. The classification result is shown in Figure 8a. The average classification accuracy of all participants keeps a high level of around 88%. This illustrates that the gestures for the same word have distinguishable common features even from different people or different sentences. The total confusion matrix of these 20 words from 3 participants is shown in Figure 8b. The word “finish” is easy to be recognized as the word “sad”, and the word “you” is also confused with “I” and “we”. This is because these two groups of words have similar hand shapes, but the hand movements are different.

#### 4.1.2. Sentence-Level User-Independent Validation

Participant 2 contributed 1000 pieces of data is regarded as the testing set in the sentence-level user-independent validation. The sign language recognition model introduced in Section 2.2.1 is applied here to only recognize a sequence of gestures without considering the grammar rule. The model is trained with the 6400 pieces of data from the remaining two participants. The testing results of Participant 2 are listed in Table 6. The substitution errors increase a lot, indicating that the recognition ability of the model drops dramatically under the condition of fewer participants. Due to the low number of experimental participants, the model could not learn enough patterns from the training set.

### 4.2. Sequence Recognition with Encoder-Decoder Model

In the sign language recognition task of Section 2.2.1, the model recognized hand gestures for each input data frame and did the alignment between the output frames and the label words with the CTC layer. Since it is a sequence-to-sequence task, it can also be achieved by an encoder-decoder structured model. Using the same model as Section 2.2.2, the decoder can generate the sequence recognition results by time steps. The recognition results with the encoder-decoder model are shown in Table 7. All three errors increase significantly compared with the CTC-based sequence recognition model. The output from the model tends to reach the “<EOS>” earlier, leading to a large number of insertion errors. Also, the decoder could not recognize gestures correctly using the information from the encoder, causing higher substitution errors.

### 4.3. Advantages and Limitations

Compared with vision-based methods, wearable sensors can obtain more direct human movement data. Recognition of sign language videos can be affected by illumination, background environment, and occlusions, but this problem will not happen while using the sensors’ data. Moreover, the real-life application of the camera involves the security of personal information and will inevitably record information about other people in the surrounding area. Most currently existing vision-based datasets are RGB videos without considering depth information. In contrast, the motion capture system restores the movement of the human body in three-dimensional space.

The proposed method has certain limitations and spaces for improvement. In this research, the movements of 300 ASL sentences are captured by the motion capture system and recognized by the deep learning model. However, it is still far away from being applied in everyday life. In addition to the limited number of sign language sentences, more external factors, such as the environment, need to be considered. The sequence recognition model can recognize each gesture with high accuracy. However, the accuracy drops in the end-to-end translation due to the difference in grammar between sign language and spoken language. A language model is needed to make the output sentences closer to spoken language. In sentence-level user-independent validation, the accuracy dropped dramatically due to the inter-individual differences in movement. More participants should be involved to let the model learn more patterns from a wide variety of data. The off-the-shelf motion capture system contains multiple inertial sensors. With the development of wearable sensor technology, more convenient devices could be considered for the research on sign language translation.

## 5. Conclusions

This research presented the basic process of sign language recognition and end-to-end translation by wearable sensors. An ASL dataset with 300 sentences for daily use was collected by the inertial motion capture system. Two kinds of deep learning models were constructed based on the grammar rule of both sign language and spoken language. Generally, the sequence recognition model achieved relatively high accuracy without considering individual differences. In contrast, the end-to-end translation presented more errors due to the lack of grammar knowledge. In the user-independent validation, the selected dataset with limited words showed high classification accuracy. In the sentence-level validation, the vocabulary containing more words increased the recognition difficulty, and the accuracy rate decreased.

## Figures and Tables

**Table 1:** The summary of previous studies on sign language recognition.

Reference	Wearables	No. of Words	No. of Sentences	No. of Participants
[6]	Myo armband	70	100	15
[7]	Smartwatch	103	73	15
[8]	Myo armband & Smartwatch	100	250	15
[14]	Myo armband	26	-	10
[15]	Myo armband	48	-	1
[16]	Myo armband	30	-	10
[19]	EMG & IMU sensors	150	-	8
[20]	EMG & ACC sensors	121	-	1
[24]	Customized data glove	26	-	3
[25]	Smartwatch & Smart ring	100	-	10
[26]	Speaker & Microphone	42	30	8

**Table 2:** The selected coordinates for sign language recognition.

Joints	Coordinates
Right & Left Arm	Rotation_Y, Rotation_X, Rotation_Z
Right & Left Forearm	Rotation_Y, Rotation_X, Rotation_Z
Right & Left Hand	Rotation_Y, Rotation_X, Rotation_Z
Right & Left Hand Thumb 1	Rotation_Y, Rotation_Z
Right & Left Hand Thumb 2	Rotation_Y, Rotation_Z
Right & Left Hand Thumb 3	Rotation_Y
Right & Left Hand Index 1	Rotation_Z
Right & Left Hand Index 2	Rotation_Z
Right & Left Hand Middle 1	Rotation_Z
Right & Left Hand Ring 1	Rotation_Z
Right & Left Hand Pinky 1	Rotation_Z

**Table 3:** The sign language recognition results.

Fold	No. of Words	No. of Sentences	Insertion Errors	Deletion Errors	Substitution Errors	WER	SER
1	5552	1480	1	13	27	0.74%	2.09%
2	5616	1480	6	10	1	0.30%	1.15%
3	5566	1480	6	36	35	1.38%	3.65%
4	5585	1480	8	34	35	1.38%	3.85%
5	5621	1480	15	14	18	0.84%	2.57%
Average	5588	1480	7	21	23	0.93%	2.66%

**Table 4:** The end-to-end sign language translation results.

Fold	No. of Words	No. of Sentences	Insertion Errors	Deletion Errors	Substitution Errors	WER	SER
1	6925	1480	551	114	263	13.40%	34.19%
2	6960	1480	991	97	470	22.39%	47.03%
3	6913	1480	665	67	207	13.58%	30.95%
4	6964	1480	546	208	438	17.12%	39.93%
5	6938	1480	501	213	443	16.68%	44.26%
Average	6940	1480	651	140	364	16.63%	39.27%

**Table 5:** Word-level user-independent validation with 20 ASL words.

Word Index	0	1	2	3	4	5	6	7	8	9
Selected Word	also	church	eat	feel	finish	friend	fries	go to	happy	have
Word Index	10	11	12	13	14	15	16	17	18	19
Selected Word	I	my	sad	summer	Sunday	this	we	year	you	dislike

**Table 6:** Sentence-level user-independent validation results.

	Substitution Errors	Deletion Errors	Insertion Errors	Total Errors	No. of Words or Sentences	Error Rate	Accuracy Rate
Word-Level Evaluation	1451	360	71	1882	3400	55.36%	44.64%
Sentence-Level Evaluation	-	-	-	729	1000	72.90%	27.10%

**Table 7:** Sequence recognition result with encoder-decoder model.

	Substitution Errors	Deletion Errors	Insertion Errors	Total Errors	No. of Sentences or Words	Error Rate	Accuracy Rate
Word-Level Evaluation	358	52	131	1480	5582	9.69%	90.31%
Sentence-Level Evaluation	-	-	-	388	1480	26.22%	73.78%

## Data Availability

The data presented in this study are available on request from the corresponding author.

## Appendix A

**Table A1:** Examples of sentences under both sign language and spoken language grammar.

Sign Language Sentences	Spoken Language Sentences
nice meet you	nice to meet you
i learn sign i	I learn sign
your name what	what’s your name
you from where	where are you from
your favorite movie what	what’s your favorite movie
you like what_do	what do you like to do
you work where	where do you work
time what	what time
bathroom where	where is bathroom
please sign again slower	please sign again slower
i have two dog one cat	I have 2 dogs and 1 cat
my favorite sport basketball	my favorite sport is basketball
i want pancake sausage	I want pancakes and sausages
meet new people my son become shy	when meeting new people my son becomes shy
you look worry you	you look worried
lion where	where is the lion
elephant huge	elephant is huge
my book where	where is my book
you like icecream you	do you like ice-cream
more soup please	more soup please
you want fries also	do you also want fries
wow steak delicious	wow the steak is delicious
happy new year	happy new year
christmas you want what you	what do you want for Christmas
tomorrow weather what	what’s the weather tomorrow
senior year i take calculus i	I take calculus in senior year
picnic i bring hotdog i	I bring hotdogs for picnic
i think future maybe become biologist	I think I may become a biologist in future
laughing commercial funny	haha the commercial is funny
sunday you want watch super_bowl you	do you want to watch super bowl on Sunday

**Table A2:** The sign language recognition vocabulary.

AC, AUG, US, a_little, a_lot, across_street, afraid, afternoon, again, age, alcohol, allergic, alright, also, altogether, always, angry, animal, annoy, apartment, apple, area, arrive, baby, back, bad_at, bake, baseball, basketball, bathroom, beach, because, become, before, biologist, birthday, black, blonde, blood, book, borrow_me, boxing, bring, broke, brother, brown, brush_teeth, busy, but, cake, calculus, canada, car, cash, cat, celebrate, chair, chemistry, chocolate, christmas, christmas_day, church, class, cleaning, clinic, close, clothes, cloud, coat, coffee, cold, college, color, come, come_on, comfortable, commercial, compete, congratulations, cook, cookies, cost, costume, country, cousin, credit, cute, dad, dark, daughter, day, deaf, delicious, department, dessert, different, dinner, dislike, do_you, dog, dollars, drink, drug, dry, due, during, easter, eat, eat_breakfast, egg, eight, elephant, engineering, english, enjoy, every_day, every_monday, every_morning, every_saturday, every_thursday, excited, excuse_me, experience, fall, family, fancy, favorite, feel, fifteen, fine, finger_spell, finish, five, five_oclock, flower, food, football, for, forget, friday, friend, fries, from, funny, future, game, get_together, ghost, give_me, go, go_ahead, go_out, good, good_at, goodbye, graduate, grandma, growing_up, hail, hair, half_time, halloween, hamburger, happy, hat, hate, have, hawaii, he, hearing, hearing_aid, heart, hello, help, help_you, here, hey, high, hiking, history, holiday, home, hope, host, hot, hotdog, hotel, house, how, how_many, how_old, huge, hungry, hunt, husband, i, icecream, important, introduce, jingle_bells, join, karate, ketchup, knit, know, lake, language, large, last, latter, laughing, learn, leave, lets, light, like, lion, live, local, look, look_around, lost, love, love_all, love_it, lunch, machine, major, make, manage, many, marry, maybe, medicine, medium, meet, meeting, milk, mix, monday, money, monkey, more, morning, mother, mountain, move, movie, museum, mustache, my, name, nauseous, need, nervous, new, next_to, next_week, nice, night, nine, nine_oclock, nineteen, no, not, not_know, not_yet, now, office, olympics, one, optometrist, orange, order, other, our, pack, pain, pancake, paper, parade, party, pass_out, pay, people, physics, picnic, pineapple, pink, plan, play, please, police_officer, potato, prayer, pregnant, president, pressure, principal, problem, pumpkin, purse, rabbit, rain, rainbow, read, ready, red, reduce, remember, resolution, rest, restaurant, run, run_out, sad, same, saturday, sausage, scared, scarf, school, season, seatbelt, see, seizures, senior, seven, seven_dollars, she, shirt, shop, show, shrimp, shy, sign, since, sister, six_oclock, skill, slower, small, snow, soda, sometimes, son, soup, spaghetti, spider, sport, spread, spring, start, state, stay, steak, still, strawberry, strong, student, study, subscribe, summer, sunday, sunny, super_bowl, support, sweet, swimming, take, take_care, take_out, talkative, teacher, team, tend, test, thank_you, thankful, thanksgiving, thanksgiving_day, that, they, think, thirty, this, three, three_oclock, three_of_you, thursday, time, tomorrow, tornado, trash, travel, treat, trick, try, turkey, turn_on, twelve_oclock, twenty_six, two, two_of_us, two_week, uncle, understand, use, vacation, valentine, vegetable, visit, vote, waitress, walk, want, warm, wash, watch, we, wear, weather, weekend, welcome, what, what_do, what_time, what_year, whats_up, when, where, which, white, who, whole, why, wife, win, winter, witch, with, work, working, workout, worry, wow, wrestling, write, year, yes, you, you_all, you_mind, your, yourself.

**Table A3:** The sign language translation vocabulary.

1, 12, 15, 19, 2, 26, 3, 30, 31, 35, 5, 6, 7, 87, 9, :, AC, August, Canada, Christmas, Easter, English, Friday, Halloween, Hawaii, I, Monday, Saturday, Sunday, Thanksgiving, Thursday, US, Valentine’s, a, across, afraid, afternoon, again, age, ahead, alcohol, all, allergic, alright, also, altogether, always, am, an, and, angry, animal, annoyed, apartment, apple, are, area, arrive, as, at, baby, back, bad, bake, baseball, basketball, bathroom, be, beach, because, become, becomes, before, bells, biologist, birthday, birthdays, black, blonde, blood, book, borrow, both, bowl, boxing, breakfast, bring, bringing, broke, brother, brown, brush, busy, but, cakes, calculus, car, care, cash, cat, celebrating, chair, chemistry, chocolate, church, class, cleaning, clinic, close, clothes, clouds, cloudy, coat, coffee, cold, college, color, come, comfortable, coming, commercial, competing, congratulations, cooked, cookies, cooking, cost, costs, costume, country, cousin, credit, cute, dad, dark, daughter, day, deaf, delicious, department, dessert, did, different, dinner, dislike, dislikes, do, does, dog, dogs, dollars, drinking, drugs, dry, due, during, eat, eating, eggs, elephant, engineering, enjoy, enjoys, every, everyday, excited, excuse, experience, fall, family, fancy, favorite, feel, fine, finger, finish, finished, flowers, food, football, for, forget, friend, friends, fries, from, funny, future, game, get, ghost, give, go, going, good, goodbye, graduate, graduation, grandma, growing, had, haha, hail, hair, half, hamburgers, happy, has, hat, hate, have, hearing, hearing-aid, heart, hello, help, here, hey, high, hiking, him, his, history, holidays, home, hope, host, hot, hotdogs, hotel, house, how, huge, hungry, hunting, husband, ice-cream, important, in, introduce, is, it, jingle, join, karate, ketchup, knit, know, lake, language, large, last, latter, learn, leave, let’s, light, like, lion, little, live, lives, local, look, lost, lot, love, lunch, machine, major, make, manage, many, marry, may, me, medicine, medium, meet, meeting, merry, milk, mind, mix, mom, money, monkey, more, morning, mother, mother’s, mountain, moved, movie, movies, much, museum, mustache, my, name, nauseous, need, needed, nervous, new, next, nice, night, no, not, now, of, office, officer, old, olympics, on, optometrist, or, orange, order, other, others, our, out, packing, pain, pancakes, paper, parade, party, pass, pay, people, physics, picnic, pineapple, pink, plan, play, please, police, potato, prayer, prefer, pregnant, president, pressure, principal, problem, pumpkin, purse, rabbits, rain, rainbow, rains, rainy, read, ready, red, reduction, remember, resolution, restaurant, resting, running, sad, same, sausages, scared, scarf, school, season, seatbelt, see, seizures, senior, shirt, shop, show, shrimps, shy, sign, since, sister, skills, slower, small, snow, soda, sometimes, son, soup, spaghetti, spell, spider, sport, spread, spring, start, starts, state, states, stay, steak, still, strawberry, street, strongly, student, studying, subscribe, summer, sunny, super, support, sweet, swimming, take, talkative, teacher, team, teeth, tends, test, thank, thankful, that, the, there, they, think, this, three, time, to, today, together, tomorrow, too, tornado, trash, travel, treat, trick, try, turkey, turning, two, uncle’s, understand, up, use, vacation, vegetable, visit, vote, waitress, walking, want, wants, warm, wash, watch, watching, we, wear, weather, week, weekends, weeks, welcome, what, what’s, when, where, which, white, who, whole, whose, why, wife, will, win, winter, witch, with, work, working, workout, worried, would, wow, wrestling, write, year, yes, yet, you, your, yourself, <BOS>, <EOS>.
